# Middleborns Disadvantaged? Testing Birth-Order Effects on Fitness in Pre-Industrial Finns

**DOI:** 10.1371/journal.pone.0005680

**Published:** 2009-05-25

**Authors:** Charlotte Faurie, Andrew F. Russell, Virpi Lummaa

**Affiliations:** 1 Department of Animal and Plant Sciences, University of Sheffield, Sheffield, United Kingdom; 2 Institute of Evolutionary Sciences, University of Montpellier, Montpellier, France; 3 Section of Ecology, Department of Biology, University of Turku, Turku, Finland; London School of Economics, United Kingdom

## Abstract

Parental investment is a limited resource for which offspring compete in order to increase their own survival and reproductive success. However, parents might be selected to influence the outcome of sibling competition through differential investment. While evidence for this is widespread in egg-laying species, whether or not this may also be the case in viviparous species is more difficult to determine. We use pre-industrial Finns as our model system and an equal investment model as our null hypothesis, which predicts that (all else being equal) middleborns should be disadvantaged through competition. We found no overall evidence to suggest that middleborns in a family are disadvantaged in terms of their survival, age at first reproduction or lifetime reproductive success. However, when considering birth-order only among same-sexed siblings, first-, middle- and lastborn sons significantly differed in the number of offspring they were able to rear to adulthood, although there was no similar effect among females. Middleborn sons appeared to produce significantly less offspring than first- or lastborn sons, but they did not significantly differ from lastborn sons in the number of offspring reared to adulthood. Our results thus show that taking sex differences into account is important when modelling birth-order effects. We found clear evidence of firstborn sons being advantaged over other sons in the family, and over firstborn daughters. Therefore, our results suggest that parents invest differentially in their offspring in order to both preferentially favour particular offspring or reduce offspring inequalities arising from sibling competition.

## Introduction

In species with altricial young, parental investment has profound effects on offspring survival [1], [2] and success [3]–[5]. However, parental investment is not unlimited [6], [7] and offspring quality is seldom equal, potentially leading to selection pressures on parents to invest differentially in their offspring [6], [8], [9]. The two most common ways in which offspring are suggested to receive greater resources is: (a) through direct competition with each other; and (b) through differential allocation of parental investment [10], [11]. These two alternatives are not mutually exclusive, and parents might invest so as to either increase or decrease levels of sibling competition [12]. For example, greater investment in certain offspring or longer birth intervals can cause inequalities between siblings. Moreover, parental investment strategies and offspring outcomes can also depend on the resource availability to parents or, in humans, in socio-economic factors [13]–[15].

**Figure 1:**
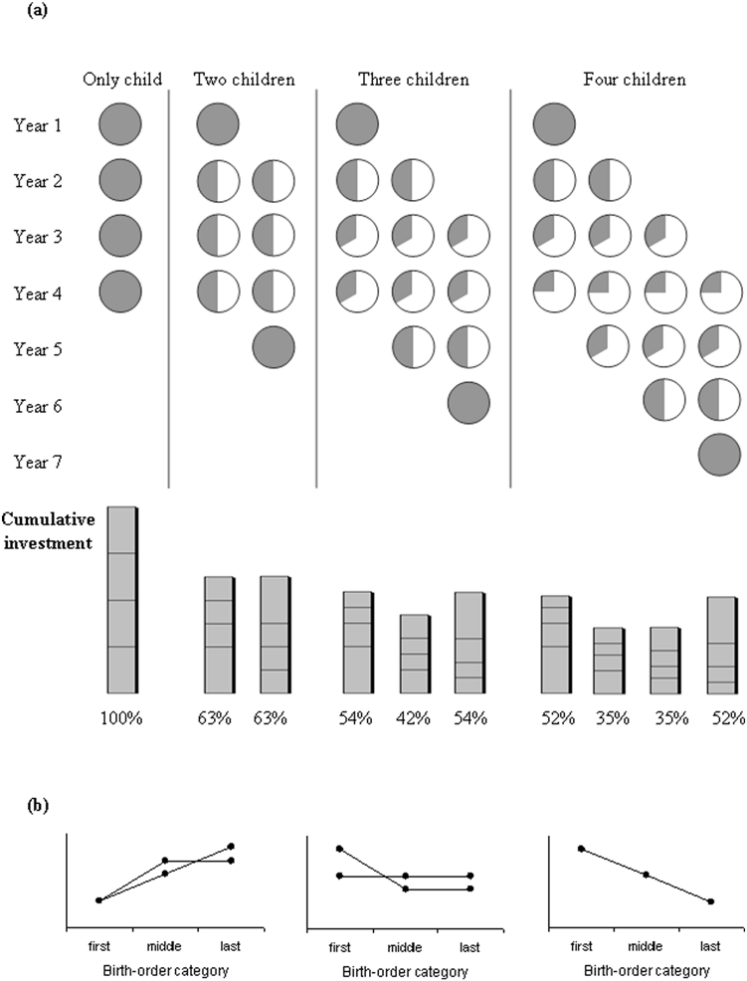
Alternative predictions regarding fitness effects of birth-order. (a) Resource allocation according to the equity heuristic as a function of birth rank in families with one to four children. Spheres in the upper part of the figure represent the proportion of resources obtained for a child in each of several birth ranks, in sibship sizes ranging from one to four, and across seven periods of growth (‘years’). The bars in the lower part show the cumulative resource distribution for children in an only-child, a two-child, a three-child and a four-child family. Whereas an equity motive produces a fair distribution at any given point in time, it yields a cumulative distribution of investments that is unequal. Adapted with permission from Hertwig, Davis & Sulloway (2002). (b) Fitness as a function of birth-order category (first-, middle- and last-borns): three example alternative predictions to that posited by Hertwig *et al.* (2002). Left panel: if parental experience is important for offspring success, or if parental investment increases as expected residual reproductive value decreases, all else being equal, middleborns and lastborns should be at an advantage. Middle panel: if parents favour firstborns (from which fitness benefits are likely to be greatest), a higher fitness is expected for firstborns compared with other categories; if parents favour middleborns which are likely to be at a competitive disadvantage, similar success is expected irrespective of birth-order category. Right panel: if resources diminish over time, lastborns are expected to be at a disadvantage.

In humans, parents need to divide their limited resources between several simultaneously dependent offspring [16], [17]. While simultaneously raising several offspring of different ages and developmental stages is typical in humans, such investment patterns can also occur in other mammals [e.g. meerkats *Suricata suricatta*: 18]; [kangaroos *Macropus* spp.: 19]. How parents allocate their investment to each offspring at each given time across their lifespan is not well-known, and few studies have investigated in any species how parents should allocate resources among offspring of different ages [20], [21]. Hertwig, Davis and Sulloway showed that if parents subdivide the resources equally among their offspring at any given point in time, it yields a cumulative distribution of investment that is unequal among offspring [22] (the “equity heuristic”, see Figure 1a). The model of Hertwig *et al.* provides a useful null hypothesis against which parental investment tactics can be measured [22]. If parents do not differentially invest in offspring and if resources do not change over time, then we would predict that firstborns and lastborns will be more successful than middleborns which, because of competition, necessarily receive less care (see Figure 1a). By contrast, in cases where parents differentially invest resources to particular offspring we would predict that the pattern of Hertwig *et al.* will not be upheld. For example, if parental experience is important for offspring success, or if parental investment increases as expected residual reproductive value decreases [23], [24], all else being equal, middleborns and lastborns should be at an advantage (Figure 1b, left hand panel). At a given time, however, the potential fitness gain that parents can obtain from firstborns is higher, because: (1) they are more likely to survive to adulthood, as they have already survived the first years of life where mortality is the highest; and (2) they are likely to start reproducing earlier, thereby shortening the generation time [15], [25], [26]. If parents favour firstborns from which fitness benefits are likely to be greatest or middleborns which are likely to be at a competitive disadvantage, we would predict greater success among firstborns compared with other categories or similar success irrespective of birth-order category, respectively (Figure 1b, middle panel). Finally, if resources diminish over time [27], we would expect that lastborns are at a disadvantage (Figure 1b, right hand panel). We acknowledge that the predictions from each of the above alternatives are not mutually exclusive and that other possibilities are equally valid, but highlight them here as example alternatives to the predictions of Hertwig *et al.* (Figure 1a).

Although there are currently no formal tests of such models, birth-order in humans is known to be associated with various child attributes. Firstborns generally have a smaller birth weight [28]. By contrast, later-born children can be out-competed for resources that are most critical in early childhood. For example, they are less likely than firstborns to attend infant clinics and to receive vaccination against childhood diseases [29], are more poorly nourished than firstborns [30], [31], and in large families, they run a higher risk of experiencing accidents during early childhood [32]. Associations between birth order and educational level have also been found; usually suggesting that firstborns are advantaged [28], [33]–[36]. Consequently, birth-order has been shown to be linked to a range of traits potentially associated with offspring fitness, such as their survival [37], wealth inheritance [38], marriage prospects [39] and number of offspring [40], [41].

However, the effects of birth order on such results are commonly contradicting. For example, some studies found that earlier-born children are at a disadvantage [e.g. 42], [43], [44], others found the opposite [e.g. 28], [29], [45], [46], at least one [47] found a U-shape mortality risk during the first nine months (elevated risk for first- and third-borns in Bangladesh) and some have found no effect at all [23]. These contradictions could be due to different balances in the relative influences of low birthweight and health in firstborns, and insufficient parental resources directed towards the health and safety of later-borns. This balance could further vary according to environmental conditions experienced by the different populations studied. Birth-order effects may also be further influenced in many humans societies by a favoured inheritance of parental possessions to first-born sons [rule of primogeniture: 38], [40], [41], [43], [48], although there are also societies where parental wealth is transferred to the youngest son [46]. Importantly, in most societies, these inheritance rules are mainly relevant to higher status families – where there is wealth to be transmitted [14].

While a number of previous studies have thus investigated birth-order effects (see above), many have failed to control for many of the potential confounding terms; which might partly account for contradictory results (see above). It thus would appear critical to control for all the factors that are known to be linked both to birth order and to survival or reproductive success. Some studies, for example, controlled for socio-economic status, but failed to control for total number of siblings or maternal age [43], [49], although this is critical when investigating birth order effects. Indeed, child mortality has been found to be negatively related to maternal age [28], and lastborns are more likely to be born from an older mother. Similarly, mothers experiencing high child mortality tend to have more births, which may make late-birth-order children appear to have higher than average mortality. In addition, no study to date has investigated the effects of birth order on a long-term measure of fitness such as the lifetime reproductive success (i.e. number of offspring raised to adulthood) that combines birth-order effects on survival, as well as reproductive rate and the quality of the offspring produced.

The aim of this study was to use the predictions of the equal parental investment model (Figure 1a) as a board to investigate incidences of differential investment on fitness and the key life-history traits underlying fitness in humans. First, we investigate the consequences of being first-, middle- or last-born on the chances of surviving to adulthood and reproducing. Second, we analyse the effects of being first-, middle- or last-born on individual lifetime reproductive success (LRS) (number of offspring raised to adulthood over lifetime) as well as the underlying components of LRS, *i.e.* lifetime fecundity (number of offspring born) and offspring survival probability. Finally, we investigate the effects of being first-, middle- or last-born on the underlying life-history traits that influence lifetime reproductive success: age at first and last reproduction and inter-birth intervals. Although the equal parental investment model (Figure 1a) explicitly considers only overall birth order (first-, middle- and lastborn child among all siblings), intra-sex birth order may, at least in some socio-ecological contexts, be more relevant to varying patterns of parental investment. Therefore, we repeated our analyses for intra-sex birth order categories (first-, middle- and lastborn son among male offspring only, and first-, middle- and lastborn daughter among female offspring only, i.e. not necessarily being first-, middle- and lastborns in the whole family). We also investigated the effects of being first-, middle-, or lastborn on a son's probability of becoming a landowner, to test the possibility that any higher success among firstborn sons may be explained by gaining access to higher resources at reproductive ages.

We use demographic pedigree records of over 2000 families from four parishes of rural Finland in the 18^th^ and 19^th^ centuries [50]. Our demographic data has at least four benefits for addressing our aims. Firstly, it was collected from church registers maintained since the 17^th^ century in each parish of the country by local clergymen, who were obliged by law to submit accurate records of the survival and reproductive history of all individuals in their parish area to the state [51]. Migration rates were low and in most cases the parish migration registers allow the lifetime reproductive success of dispersers to be determined. Secondly, the study period ends before the availability of reliable contraception, freely available healthcare and the associated transition to low mortality and fertility in Finland, which was not complete until the mid-20^th^ century [52]. Hence, mortality and fertility rates are close to ‘natural’. Thirdly, the data includes the social status of each family, which represents differences in resource availability in terms of nutrition, wealth and workload between individuals, and has already been shown to influence reproductive success in our study population [53], [54] as well as other pre-industrial populations [e.g. 14]. Fourth, our data allows a number of other confounding factors to be controlled, including geographic (study parish) and temporal (birth cohort) variation in fertility and mortality, as well as number of siblings, maternal age and maternal survival.

## Methods

This study was based on demographic records from historical Finnish populations. Our data contain three generations of pedigree data for four geographically separate rural parishes from the south-west archipelago and from the mainland (Hiittinen, Kustavi, Rymättylä, and Ikaalinen). During the study period, these populations depended on farming for their livelihood, supplemented with fishing in the archipelago areas of south-western Finland. Farmers could either own their land, or rent it (tenant farmers). Information on the occupation of each man, or for women, that of their husband, allowed us to control for socio-economic status. We contrasted two groups, according to those owning land versus those either renting or having no access to land at all. Importantly, these categories have already been associated with variation in individual life-history traits including fitness among humans living in pre-industrial conditions [46], [55]. The mating system was monogamous. Divorce was forbidden, and so remarriage was permitted only in the event of spousal death. Child mortality was high, with only 62% of individuals in our sample surviving to age 15 (the youngest age of first reproduction recorded in this population). During the study era, inheritance usually favoured the firstborn son (rule of primogeniture), although inheritance practices varied in time and place [56]. In most parishes, the firstborn son inherited the farm (but in the archipelago parishes, the firstborn daughter could sometimes inherit the farm). Concerning capital, cattle and personal property, all male offspring were granted equal shares, while daughters received a half share [57]. The firstborn son who inherited the farm was obliged to pay his brothers and sisters their due, but the compensation was often lower than the value of a share in the farm and land. The bride's dowry consisted of personal property (e.g. tools, clothes, bed, chest), head of cattle (cows, sheep), and money [58].

The study sample includes 2180 men and 2126 women (F1 generation) born between 1732 and 1882 to 716 mothers (P generation). We recorded full life-history data (survival and lifetime reproductive events) for all F1 individuals, and followed their offspring (F2) until age 15. Birth-order from the mother's point of view was used to build the variable of interest, comprising three categories: firstborns, middleborns and lastborns. Twins were excluded from the sample, as were families of less than three children because the middleborn resource handicap can only be considered in families of three or more children (Fig 1) [22]. The resulting sample of generation F1 (3,616 individuals) comprised 577 firstborns (310 males and 267 females), 2,459 middleborns (1,260 males and 1,199 females), and 577 lastborns (274 males and 303 females). For intra-sex birth order analyses, the male subsample (1509 men who had at least two brothers) comprised 350 firstborn sons, 809 middleborn sons, and 350 lastborn sons, and the female subsample (1417 women who had at least two sisters) comprised 344 firstborn daughters, 729 middleborn daughters, and 344 lastborn daughters.

Statistical analyses used to address our questions were conducted using SAS (SAS Institute Inc., release v. 9.1, 2002–2003). In all analyses, the following variables were entered into a model to control for potentially confounding sources of variation: sex; mother's age at birth; mother's survival (age at mother's death); number of siblings; parental socio-economic status (landowners *vs.* landless); geographic area (mainland vs. archipelago); birth cohort (5 categories with 20 years blocks). In all models, the identity of mothers was fitted as a random term to account for the use of several offspring within families. Statistically significant terms at the level of 0.05 were determined. Once the minimal model was found, birth-order category (our term of interest) was added and its significance determined. All biologically meaningful two-way interactions involving birth-order (with sex, socio-economic status, number of siblings, and maternal age) were also tested but only included and reported here if statistically significant.

The proportional response variables (probabilities of surviving to adulthood and reproducing; survival probability of all children produced) were analysed with Generalised Linear Mixed Model (GLMM) where the response term was fitted to a binomial error structure and a logit link function (using GENMOD function in SAS). For binary response terms, binomial denominator was fixed at 1. For the proportion of offspring surviving to adulthood, the number of surviving offspring (LRS) was considered as the response term with a variable binomial denominator equal to the number of offspring born (fecundity). The count response variables (LRS and fecundity) were analysed with GLMM where the response term was fitted to a Poisson error structure and a logarithm link function (using GENMOD function in SAS). The continuous response variables (age at first and last reproduction, length of birth intervals) were analysed with linear mixed-effects models (LME, using MIXED function in SAS) with the response terms being fitted to a normal error structure and Satterthwaite's formula being used to approximate the denominator degrees of freedom of each fixed effect [59].

### Probability of surviving to adulthood and reproducing

To determine whether birth-order affected survival probability to adulthood (age 15), we included all individuals who died before age 15 or were successfully followed at least until age 15 (3,583 individuals, 99% of our initial sample). For the probability of reproducing, we restricted our analyses to individuals who survived to age 15 and who had been successfully followed at least until the age at which 90% of the individuals in the population had already finished reproducing, if they were to ever reproduce in their lifetime (age 50 for men and 44 for women). If the individual disappeared after this age and had not reproduced before, he/she was considered as having never reproduced. The subset of individuals for which reproductive events were known comprised 843 men and 913 women (77% of individuals who survived to adulthood). These individuals did not differ from those excluded from the sample in the proportions of first-, middle- and lastborns (16%, 68% and 16% in both subsets).

### Lifetime reproductive success (LRS)

LRS was measured as the lifetime number of F2 offspring raised to 15 years. We restricted the sample to include only those individuals who had been successfully followed at least until the age at which 90% of the individuals in the population had already finished reproducing (age 50 for men and 44 for women), for which LRS is known, and who reproduced at least once (739 men and 794 women).

### Components of LRS

Two life-history traits will govern our measure of LRS: lifetime fecundity (number of offspring born) and the survivorship of offspring to adulthood. Lifetime fecundity was considered as a count response term. The proportion of offspring surviving to age 15 was examined by considering LRS as a response term in a GLMM with logit link function and a variable binomial denominator equal to fecundity. For these analyses, we used the same sample as for LRS.

### Underlying life-history traits and probability of landowning

We examined more specific life-history traits involved in LRS: age at first reproduction, age at last reproduction, and average inter-birth interval (*i.e.* length of reproductive life divided by the number of children). For the analysis of inter-birth interval, we used the sub-sample of individuals who reproduced at least twice (694 men and 731 women). Finally, among men who survived to adulthood, we investigated whether being a first-, middle- or lastborn son influenced socio-economic status, i.e. whether they owned a land. The sample of families with at least three sons, for which both own and parental socio-economic status were known, comprised 947 men.

## Results

### Probability of surviving to adulthood and reproducing

**Figure 2:**
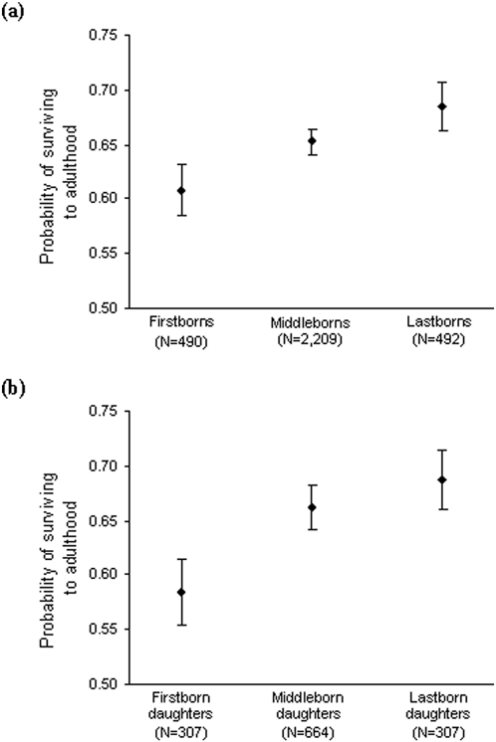
Probability of surviving to adulthood (age 15). (a) As a function of overall birth-order category: the probability of surviving to adulthood was marginally significantly influenced by whether an individual was a firstborn, middleborn or lastborn among all offspring (χ^2^_2_ = 5.76, *p* = 0.056). (b) As a function of intra-sex birth-order category (among daughters): the probability of surviving to adulthood was significantly influenced by whether a daughter was a firstborn, middleborn or lastborn (χ^2^_2_ = 6.48, *p* = 0.04). Figures show predicted values of the minimal model and error bars represent standard errors of the means.

In our sample, 63.9% of F1 individuals survived to age 15 (63.3% of boys and 64.4% of girls, N = 3,583). Survival was positively associated with mother's (P generation) survival (χ^2^_1_ = 10.43, *p* = 0.001), and negatively associated with the number of siblings (χ^2^_1_ = 18.52, *p*<0.0001). After controlling for these significant effects, we found clear, although marginally non-significant, trend for differences between overall birth-order categories (all siblings of both sexes considered) in their probability of surviving to adulthood, with firstborns being disadvantaged, and lastborns having the best chances of surviving (χ^2^_2_ = 5.76, *p* = 0.056; Figure 2a). The model explained 13% of the variance.

When considering intra-sex birth-order, we did not find any significant differences between first-, middle- and lastborn sons in their probability of surviving to adulthood (respectively 62%±3%, 63%±2%, 68%±3%; χ^2^_2_ = 2.66, *p* = 0.3), but we found that firstborn daughters had a significantly reduced probability of surviving to adulthood, as compared to their sisters (χ^2^_2_ = 6.48, *p* = 0.04; Figure 2b), controlling for total number of siblings (χ^2^_1_ = 13.56, *p* = 0.0002) and for mother's survival (χ^2^_1_ = 5.17, *p* = 0.02). The model explained 12% of the variance.

Of those F1 individuals who survived to adulthood and were successfully followed until the end of potential reproductive life, 87.3% reproduced (87.7% of men and 87.0% of women, N = 1,756). Table 1 provides the descriptive statistics of this data subset split by sex and parental socio-economic status.

**Table 1:** Descriptive statistics of reproductive parameters of F1 individuals who survived to age 15 and were successfully followed until the end of potential reproductive life, split by sex and parental socio-economic status (mean±s.d.).

*SES*	*Sex*	*N*	*Lifespan*	*% Rep*	*AFR*	*ALR*	*L. Fec.*	*LRS*
Own land	M	531	53.1±17.6	87.1%	28.0±5.7	40.5±8.4	5.2±3.4	3.2±2.3
	F	519	58.2±18.8	86.0%	26.3±5.0	38.2±5.6	4.8±3.2	3.0±2.3
Landless	M	447	52.9±18.7	88.4%	28.3±5.8	41.8±9.0	5.2±3.3	3.2±2.4
	F	459	56.6±19.0	88.1%	26.5±4.6	38.0±6.2	4.5±2.9	2.7±2.1
**All**	**M**	**978**	**53.0±18.1**	**87.7%**	**28.2±5.8**	**41.1±8.7**	**5.2±3.3**	**3.2±2.3**
	**F**	**978**	**57.5±18.9**	**87.0%**	**26.4±4.8**	**38.1±5.9**	**4.7±3.1**	**2.9±2.2**

(SES: parental socio-economic status; N: sample size; % Rep: probability of reproducing; AFR: age at first reproduction; ALR: age at last reproduction; L. Fec.: lifetime fecundity; LRS: lifetime reproductive success).

We found no evidence to suggest that probability of reproducing in a lifetime depended on birth-order category among all siblings (respectively 89%±2%, 87%±1%, 88%±2%; χ^2^_2_ = 1.39, *P* = 0.5), or among brothers only (respectively 87%±3%, 86%±2%, 88%±3%; χ^2^_2_ = 0.23, *P* = 0.9), or among sisters only (respectively 90%±3%, 86%±2%, 87%±3%; χ^2^_2_ = 1.42, *P* = 0.5).

### Lifetime reproductive success (LRS)

Among F1 individuals who survived to age 15, who were successfully followed until the end of their potential reproductive life, and who reproduced at least once, the average LRS was 3.3±2.2 s.d., and the maximum was 11. LRS differed between geographical areas (χ^2^_1_ = 13.30, *p* = 0.0003), and between cohorts (χ^2^_4_ = 20.36, *p* = 0.0004). In this subset of individuals who did reproduce, men had more children than women (χ^2^_1_ = 6.96, *p* = 0.008). LRS was positively associated with P mother's survival (χ^2^_1_ = 10.90, *p* = 0.001). After controlling for these effects, we found no evidence to suggest that LRS depended on birth-order category among all siblings (χ^2^_2_ = 0.83, *p* = 0.7).

**Figure 3:**
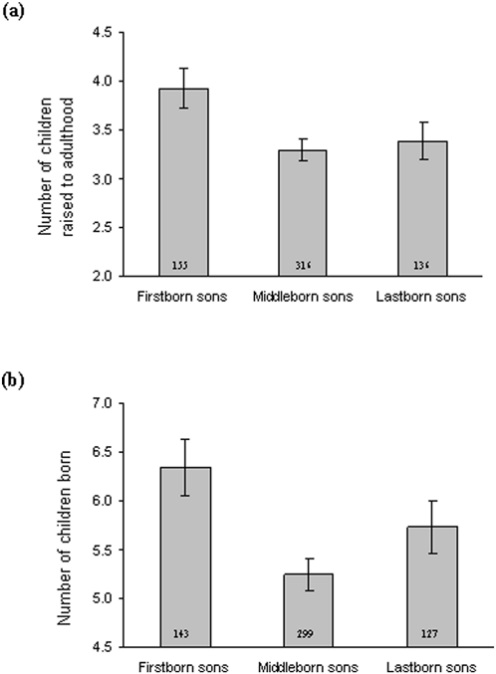
Reproductive success. (a) Lifetime reproductive success (LRS: number of children raised to age 15) as a function of intra-sex birth-order category (among sons): firstborn sons had the highest LRS, as compared to their younger brothers (χ^2^_2_ = 5.26, *p* = 0.07). No such effect was found among sisters (χ^2^_2_ = 1.60, *p* = 0.45, not shown in the figure). (b) Lifetime fecundity (number of children produced) as a function of intra-sex birth-order category (among sons): firstborn sons had the highest fecundity, as compared to their younger brothers (χ^2^_2_ = 12.91, *p* = 0.002). No such effect was found among sisters (χ^2^_2_ = 1.33, *p* = 0.5, not shown in the figure). Figures show predicted values of the minimal model, error bars represent standard errors of the means and sample sizes are indicated at the bottom of the bars.

When considering intra-sex birth-order categories, we found that first-, middle- and lastborn daughters did not differ for LRS (2.9±0.2, 3.3±0.1, 3.3±0.2; χ^2^_2_ = 2.76, *p* = 0.3). However, first-, middle- and lastborn sons did differ (χ^2^_2_ = 7.41, *p* = 0.02), controlling for total number of siblings (χ^2^_1_ = 8.48, *p* = 0.004): firstborn sons were strongly advantaged (Figure 3a). The model explained 8.6% of the variance.

### Components of LRS

Among F1 individuals who survived to age 15, who were successfully followed until the end of their potential reproductive life, and who reproduced at least once, the average lifetime fecundity (number of children born) was in our sample 5.4±3.0 s.d. (N = 1,533) and the maximum was 16. Lifetime fecundity differed between geographical areas (χ^2^_1_ = 39.69, *p*<0.0001) and between birth-cohorts (χ^2^_4_ = 9.29, *p* = 0.05). In this subset of individuals who did reproduce, men had more children than women (χ^2^_1_ = 7.44, *p* = 0.006). Lifetime fecundity was also positively associated with P mother's survival (χ^2^_1_ = 12.86, *p* = 0.0003) and with the number of siblings (χ^2^_1_ = 4.26, *p* = 0.04). After controlling for these effects, we found no evidence to suggest that lifetime fecundity was associated with overall birth-order category (5.5±0.2, 5.4±0.1, 5.7±0.2; χ^2^_2_ = 0.88, *p* = 0.6).

However, when investigating the effect of intra-sex birth-order categories, we found that firstborn sons produced significantly more offspring than their brothers and that middleborn sons produced the smallest number of offspring (χ^2^_2_ = 12.91, *p* = 0.002; Figure 3b), controlling for area (χ^2^_1_ = 8.97, *p* = 0.003), total number of siblings (χ^2^_1_ = 15.15, *p*<0.0001) and mother's survival (χ^2^_1_ = 6.54, *p* = 0.01). This model explained 17% of the variance. Additionally, we found that when each adult son's own socio-economic status was included in the model (χ^2^_1_ = 2.64, *p* = 0.1), this effect of intra-sex birth-order category on their fecundity was still significant (χ^2^_2_ = 12.28, *p* = 0.002), suggesting that that the favoured position of first-borns sons as the inheritors of the parental land was not the sole reason for their higher lifetime fecundity. No differences were found between sisters' birth order categories for fecundity (respectively 4.9±0.2, 5.2±0.2, 5.2±0.2; χ^2^_2_ = 1.33, *p* = 0.5).

On average, 62% of offspring (F2) survived to adulthood (N = 1,533). This percentage differed between geographical areas (χ^2^_1_ = 6.08, *p* = 0.01), between birth-cohorts (χ^2^_4_ = 19.26, *p* = 0.0007), and increased with socio-economic status (χ^2^_1_ = 3.13, *p* = 0.07). In addition, it was negatively associated with the number of siblings (χ^2^_1_ = 11.96, *p* = 0.0005). After controlling for these effects, we found no evidence to suggest that the proportion of an individual's offspring surviving to adulthood differed according to his/her overall birth-order categories in a family (respectively 63%±2%, 62%±1%, 61%±2%; χ^2^_2_ = 1.01, *p* = 0.6) or intra-sex birth-order categories (among brothers: respectively 60%±2%, 61%±2%, 61%±2%; χ^2^_2_ = 0.16, *p* = 0.9; among sisters: respectively 58%±2%, 62%±2%, 62%±2%; χ^2^_2_ = 1.80, *p* = 0.4).

### Underlying life-history traits and probability of landowning

**Figure 4:**
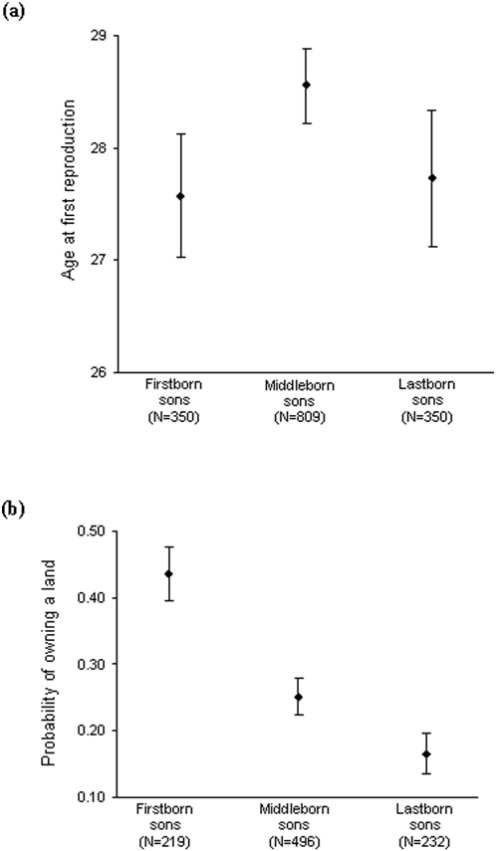
Firstborn sons start reproducing earlier, probably due to their greater access to resources. (a) Age at first reproduction as a function of intra-sex birth-order category: middleborn sons began reproduction later than firstborn sons (F_2,560_ = 6.54, *p* = 0.002). No such effect was found among sisters (F_2,564_ = 0.78, *p* = 0.5, not shown in the figure). (b) Proportion of men who owned a land among first-, middle- and lastborn sons: firstborn sons were twice more likely to become landowners (χ^2^_2_ = 25.71, *p*<0.0001).

The average age at first reproduction among F1 individuals differed between men and women (F_1,1439_ = 48.88, *p*<0.0001; see Table 1). Age at first reproduction also differed between geographic areas (F_1,1412_ = 8.13, *p* = 0.005) and was positively associated with maternal age (P generation) (F_1,1471_ = 5.98, *p* = 0.01). After controlling for these effects, we found that age at first reproduction did not significantly depend on overall birth-order category (respectively 27.5±0.4, 27.4±0.2, 26.4±0.4; F_2,1415_ = 2.60, *p* = 0.074). However, when considering intra-sex birth-order categories, we did find that firstborn sons began reproduction on average almost two years earlier than their brothers (F_2,560_ = 6.54, *p* = 0.002; Figure 4a); in contrast, age at first reproduction did not significantly differ among sisters (respectively 26.2±0.4, 26.4±0.3, 25.9±0.4; F_2,564_ = 0.78, *p* = 0.5).

The average age at last reproduction differed between men and women (F_1,1462_ = 54.29, *p*<0.0001; see Table 1). Age at last reproduction also differed between geographic areas (F_1,380_ = 18.22, *p*<0.0001) and was positively associated with mothers' survival (F_1,472_ = 11.14, *p* = 0.0009). After controlling for these effects, we found no evidence to suggest that age at last reproduction depended on overall birth-order categories (respectively 38.6±0.5, 39.4±0.2, 39.1±0.5; F_2,1418_ = 1.27, *p* = 0.3) or intra-sex birth-order categories (among brothers: respectively 40.1±0.7, 40.5±0.5, 41.6±0.7; F_2,563_ = 1.23, *p* = 0.3; among sisters: respectively 37.1±0.5, 38.0±0.4, 37.4±0.5; F_2,574_ = 1.24, *p* = 0.3).

Finally, among individuals who reproduced at least twice, average inter-birth interval (*i.e.* the length of reproductive life divided by the number of children produced) differed between birth cohorts (F_4,887_ = 2.83, *p* = 0.02) and depended on parental socio-economic status (F_1,448_ = 6.22, *p* = 0.01), but we found no difference between overall birth-order categories in a family (respectively 2.27±0.06, 2.32±0.04, 2.32±0.07; F_2,1348_ = 0.27, *p* = 0.8 or between intra-sex birth-order categories (among brothers: respectively 2.18±0.09, 2.33±0.06, 2.37±0.09; F_2,500_ = 1.38, *p* = 0.3; among sisters: respectively 2.31±0.07, 2.30±0.06, 2.29±0.08; F_2,530_ = 0.02, *p* = 0.9).

Among men, own socio-economic status as an adult was strongly positively associated with parental socio-economic status (χ^2^_1_ = 131.08, *p*<0.0001). After controlling for this effect, we found that intra-sex birth-order category also had a significant effect, with firstborn sons being much more likely than their brothers to become landowners (χ^2^_2_ = 26.47, *p*<0.0001; Figure 4b). This model explained 23% of the variance.

## Discussion

Parental investment has profound effects on offspring success [3], [4], [60]. Given that parents accrue differential fitness benefits from their offspring, parental investment (and particularly differential investment) tactics are currently an expanding subject of research in evolutionary ecology. Hertwig, Davis and Sulloway provided a useful first step by offering a potential null hypothesis against which observations of birth order effects on offspring success can be compared [22]. Our study investigated, in humans, differences between first-, middle- and lastborns using lifetime fitness measures, while simultaneously controlling for maternal age, total number of siblings and socio-economic status.

Hertwig *et al.* posit that all else being equal, differences in the amount and quality of parental investment received by offspring can arise even in the presence of an equity motive on the parents' side: in families of three or more children, middleborns will receive fewer resources due to competition with younger and elder siblings [22]. When considering overall birth-order in a family, we found no evidence for this idea despite using a large, detailed and representative dataset of over 2000 families living in pre-industrial Finland. Middleborn offspring did not have reduced probabilities of surviving to adulthood or reproducing, or reduced lifetime reproductive success. Indeed, they neither had reduced fecundity nor gave rise to offspring with reduced survivorship. Given the fact that (all else being equal) middleborns should be disadvantaged in humans and that yet we found little supporting evidence for this, it is likely that parents increase investment in middleborn offspring. However, it should be noted that when considering birth-order effects among males of the sibship, we found that middleborn sons gave birth to fewer offspring, and showed a tendency to have a lower LRS than their brothers. This was partly because they began reproduction on average one year later than their brothers. Middleborn daughters, within all the daughters of a sibship, did not suffer similar costs as middleborn sons.

We found some evidence that parental inexperience negatively influenced offspring survival to adulthood. Children who had the lowest chances of surviving were firstborns. This was especially true for firstborn daughters, who were less likely to survive than their sisters. Firstborns are often born smaller in humans [61], and birthweight is related to health in adulthood [37]. That we did not find reduced fitness among firstborns suggests some degree of compensatory investment following birth either directly through (grand)parents or through sibling competition.

In fact, modelling resource allocation among different-aged offspring indicates that, in nearly all circumstances, the evolutionary stable strategy is to bias investment toward older offspring [21]. In line with this idea, primogeniture (first offspring inheritance) is widespread in human populations [for a review, see 38], and firstborns tend to end up in an advantaged position during upbringing with regard to both health [29], [62] and educational achievement [34], [63]. In accordance with this, our results showed that firstborn sons were those who gave birth to the largest numbers of offspring, and this is presumably because they began reproduction earlier. This is likely mostly explained by their higher access to resources: firstborn sons were more likely to become landowners, and landownership is known to be related to reduced ages at first marriage and reproduction in this population [64]. These findings suggest that parental resources diminish over time, in contrast to the model of Hertwig *et al*.

A further limitation of the Hertwig *et al.* model is that it fails to consider the possibility that older brothers and older sisters are likely to have, at least in some socio-ecological settings, a different influence on their younger siblings' fitness. For example, in rural Gambia, children with any living sisters who were at least 10 years older than the child had lower mortality rates, whereas the presence of older brothers did not improve survival chances [42]. Among the Gabbra pastoralists, whereas the number of older brothers had a negative effect on men's reproductive success (as a result of smaller initial wealth and later age at marriage), the total number of sisters had a significant positive effect [41]. Similarly, among the Kipsigis of Kenya, men's reproductive success (number of offspring surviving to 5 years) decreased with the number of brothers and increased with the number of sisters [43]. Low [40] showed that the number of elder brothers negatively correlated with men's number of offspring in 19^th^ century Sweden. Finally, using the same dataset as the present study, Rickard *et al.* [65], [66] have shown that offspring born after a brother have lower lifetime reproductive success than those born after a sister. In line with these findings, our analyses showed that intra-sex birth-order affected fitness correlates in a different way among male and female siblings. Firstly, being a firstborn son provided a reproductive advantage, even though a firstborn son can be a middleborn in the family as a whole. This is obviously the consequence of wealth inheritance practices, as illustrated by the fact that firstborn sons were more likely to own a land than other men. We did not find evidence for such an advantage of firstborn daughters. While firstborn men breed with a richer resource-base than their younger brothers, this may not be true of females who marry out of the family. Secondly, the probability of surviving to adulthood was on average lower for firstborn daughters than for other girls, even though this group comprises both firstborns and middleborns in the family as a whole. The fact that, in contrast, survival was not significantly reduced for firstborn sons could be due to an increased effort made by parents to protect the life of their first son, especially in his early years, because at that time he is still the only son they have, and therefore the only potential heritor of their wealth.

Finally, the Hertwig *et al.* model fails to take into account differences in the timing of resource acquisition. In the ‘equal investment’ model, the resources received for the first and last birth rank are identical in quantity, but not in timing. For example, consideration of the resources received during the first two years of first- and lastborns in a family of four children (Figure 1a), illustrates this point. This difference in the timing of parental resource availability can be significant, for early conditions can dictate a whole host of future characteristics [60], [67].

In conclusion, we have shown that birth-order category in the sibship as a whole has little influence on fitness. By contrast, we found that, among male offspring in this population, firstborn sons had a significantly higher fitness than their brothers, and middleborn sons tended to have the lowest fitness. These results contrast with predictions of the ‘equal investment’ model [22], indicating that mothers preferentially invest in certain offspring. Future models which expand the ‘equal investment’ model to include sex differences and diminishing parental resources will be particularly valuable in providing predictive frameworks to studies of parental investment in viviparous animals. Our results suggest that resources acquisition by offspring in humans is not simply a consequence of sibling competition, but that parents actively provide extra resources to those offspring which are likely to be under the greatest competition, or which are the greatest fitness potential.
